# Electrospun Nanofibers for Sensing and Biosensing Applications—A Review

**DOI:** 10.3390/ijms22126357

**Published:** 2021-06-14

**Authors:** Kinga Halicka, Joanna Cabaj

**Affiliations:** Faculty of Chemistry, Wroclaw University of Science and Technology, Wybrzeze Wyspianskiego 27, 50-370 Wroclaw, Poland; kinga.halicka@pwr.edu.pl

**Keywords:** electrospinning, nanofibers, detection, biomedical compounds, metal ions

## Abstract

Sensors and biosensors have found applications in many areas, e.g., in medicine and clinical diagnostics, or in environmental monitoring. To expand this field, nanotechnology has been employed in the construction of sensing platforms. Because of their properties, such as high surface area to volume ratio, nanofibers (NFs) have been studied and used to develop sensors with higher loading capacity, better sensitivity, and faster response time. They also allow to miniaturize designed platforms. One of the most commonly used techniques of the fabrication of NFs is electrospinning. Electrospun NFs can be used in different types of sensors and biosensors. This review presents recent studies concerning electrospun nanofiber-based electrochemical and optical sensing platforms for the detection of various medically and environmentally relevant compounds, including glucose, drugs, microorganisms, and toxic metal ions.

## 1. Introduction

The development of various fields, such as medical diagnostics or environmental protection, has raised the need for analytical tools to enable the detection and monitoring of specific analytes. Sensors and biosensors meet this need, allowing highly selective and sensitive determination of countless molecules in analyzed samples.

Nanotechnology has raised a special interest among researchers and has led to the development of more sensitive sensors with better performance. Nanomaterials (NMs), including nanofibers (NFs), possess properties unobtainable in bulk materials, proving to be an interesting and desired addition to conventional sensor design techniques. The use of NMs allows to modify standard approaches, leading to higher loading capacity, smaller sample volume, and a possibility for the miniaturization of designed sensing platforms [1].

**Figure 1:**
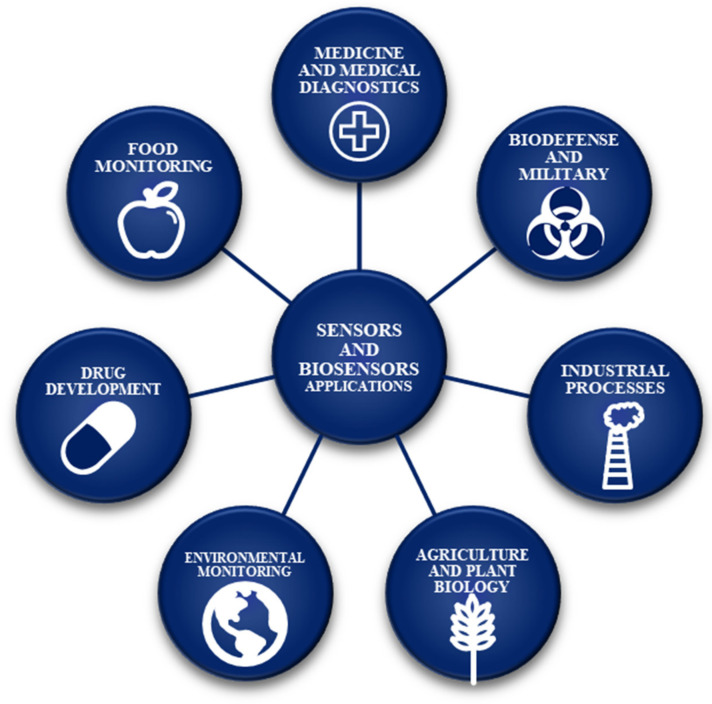
Potential applications of sensors and biosensors.

In medicine and clinical diagnostics, monitoring of compounds such as hormones, drugs, or protein markers is crucial for the prevention, diagnosis, control, and treatment of diseases. A glucometer, a sensor that measures the concentration of glucose in bodily fluids (usually in blood), remains one of the most commonly used biosensors in diagnostics, as it is essential for people suffering from diabetes [2]. Although metals are present in everyday life, in industry, and are necessary for many biological functions, e.g., for enzyme activity, some of them also pose a risk to the environment and health, which is why sensors with the ability to detect trace amounts of hazardous heavy metal ions are required [3]. Various other applications of sensors and biosensors are presented in Figure 1.

This review presents numerous examples of recently published nanofiber-based electrochemical and optical sensors and biosensors for the detection of various biomedically relevant molecules, such as glucose, neurotransmitters, and drugs, as well as for the detection of heavy metal ions.

## 2. Sensors and Biosensors

**Figure 2:**
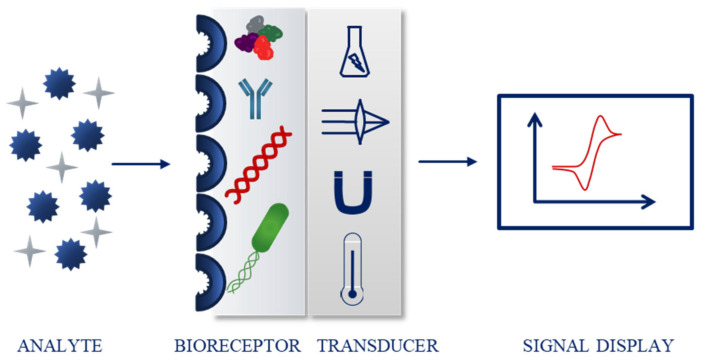
Schematic biosensor operating principle.

In 1991, the International Union of Pure and Applied Chemistry (IUPAC) defined a chemical sensor as a device capable of transforming chemical information into a signal that can be processed. The two main components of the sensor are a receptor, which changes the chemical information into a form of energy that can be measured and then transformed by the second important part, a transducer (Figure 2). Both physical and chemical principles can be the base of the receptor part, which is responsible for the recognition of the analyte. If the recognition process is based on a biochemical reaction, the sensor is considered a biosensor [4].

The most common classification of sensing devices is based on the type of transducer. Examples include optical (further divided depending on the optical properties, e.g., absorbance, fluorescence, or luminescence), electrochemical (for instance voltammetric, potentiometric), electrical, mass sensitive, thermometric, and magnetic sensors [4,5,6,7,8]. Biosensors can also be classified according to the biorecognition part. The molecules responsible for the binding of the analyte include, e.g., enzymes, nucleic acids, antibodies, bacteria, and cells (Figure 2) [4,9,10,11,12].

**Figure 3:**
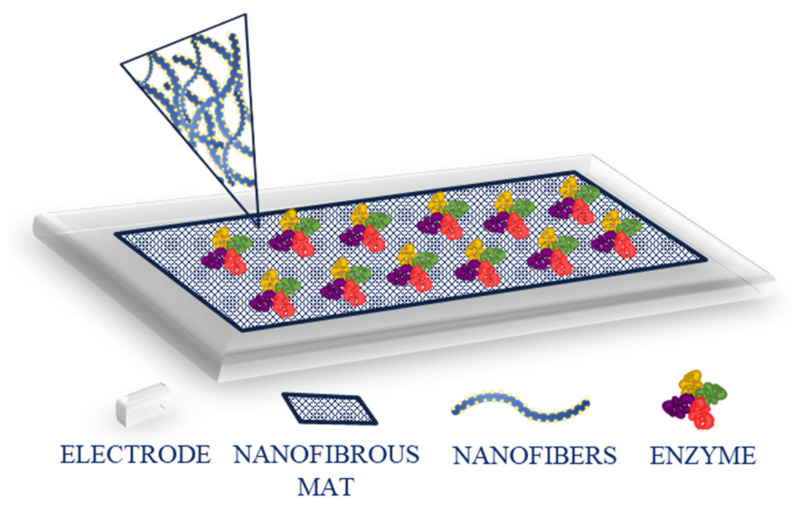
Schematic representation of enzyme immobilization on a modified electrode.

A (bio)sensor should possess several properties or characteristics that provide accurate and satisfying performance. Those include independence of physical limitations (e.g., pH or temperature), selectivity (ability to detect specific analyte from a sample), sensitivity (low limit of detection, LOD), linearity and linear range (connected to accuracy), stability (over time and in various conditions), response time, and reproducibility (repeating the experimental set-up with the same response) [13]. In the case of biosensors, the immobilization of biomolecules, such as enzymes, antibodies, and nucleic acids, is crucial for the biorecognition to occur (Figure 3).

**Figure 4:**
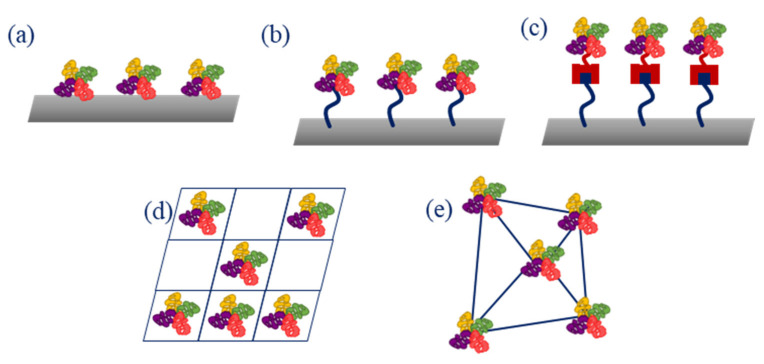
Main enzyme immobilization methods: (**a**) adsorption; (**b**) covalence bonding; (**c**) affinity bonding; (**d**) entrapment; (**e**) cross-linking.

When selecting a method of immobilization, several aspects need to be considered, as the biomolecule needs to maintain its shape, function, activity, and stability. The main methods for immobilization include entrapment of the enzyme in a 3D gel or polymeric matrix, e.g., through electropolymerization or photopolymerization, adsorption, e.g., through physical adsorption or layer-by-layer deposition, cross-linking (e.g., with glutaraldehyde), covalent immobilization that often requires activation of carboxylic and amino groups, e.g., through 1-ethyl-3-(3-dimethylaminopropyl)carbodiimide/N-hydroxysuccinimide (EDC/NHS) reaction, and affinity binding (e.g., using biotin and streptavidin affinity) (Figure 4) [14].

Among different types of sensors, two are most often described in the literature-electrochemical and optical sensors. In an electrochemical sensor, an electrical signal is produced as a result of a reaction between the matrix and the analyte. The most conventional set-up consists of three electrodes: a working electrode where the reaction occurs, a reference electrode that provides feedback, and a counter/auxiliary electrode that balances current and sets the potential of a working electrode. The most common techniques adopted in electrochemical sensors are voltammetric, amperometric, and impedimetric. In the first type, the current resulting from an electric potential (as a function of time) applied between the working and counter electrodes is measured. Common voltammetric methods are cyclic voltammetry (CV) and differential pulse voltammetry (DPV). In case of amperometry, an electric potential is applied as well, however, it’s the current that is shown as a function of time. Impedimetric sensors measure the impedance of the system. An example is electrochemical impedance spectroscopy (EIS). Advantages of electrochemical sensors include simplicity, low cost, fast response time, and miniaturization possibility [15,16]. However, some disadvantages should also be mentioned, such as environment and temperature sensitivity, requirement of redox elements, need for theoretical stimulation, or long detection time [17].

In an optical sensor, an interaction of the receptor and the analyte occurs, leading to an optical phenomenon, recorded and transformed by the device. The signal is proportional to the concentration of the analyte. Various phenomena can be adopted in optical sensors, for instance, absorbance, luminescence, fluorescence (both emission and quenching of fluorescence have been used in sensing systems), or surface plasmon resonance. Optical sensors present many advantages, such as low cost, small size, and a possibility of direct and real-time detection [4,18]. However, different types of optical sensors possess some limitations, for instance, lower sensitivity, requirement of specific equipment, and in some cases, a long detection time [19].

Nanomaterials, defined as materials with dimensions in the nanoscale, have attracted special attention in the designing of sensors and biosensors, as their properties differ from the properties of the bulk material, allowing better performance of the devices. The most important characteristic of NMs is a large surface area to volume ratio which enables better immobilization of bioreceptors. These biomolecules also benefit in terms of activity and lifetime as a result of the size comparable to that of NMs. In addition, nanostructures possess several unique properties dependent on their size and shape that will affect the design and performance of sensing platforms, including, but not limited to, optical, mechanical, electronic, and conducting properties. Various NMs have been used in sensing devices, for example, quantum dots (QDs), metal nanoparticles (NPs), nanotubes, and nanofibers, the latter being the focus of this review [1,20].

## 3. Electrospinning and Nanofibers

### 3.1. Electrospinning

The history of electrospinning can be traced back to the 17th century, when William Gilbert observed the electrostatic attraction of a liquid-a water droplet forming a cone shape in the presence of an electric field [21,22]. However, the technique itself was developed (or first observed) in 1897 by Lord Rayleigh [23,24]. It is one of the simplest and therefore most commonly used techniques for the fabrication of nanofibers. It allows to produce NFs with diameters ranging from a few nanometers to micrometers from a polymer solution [24,25].

**Figure 5:**
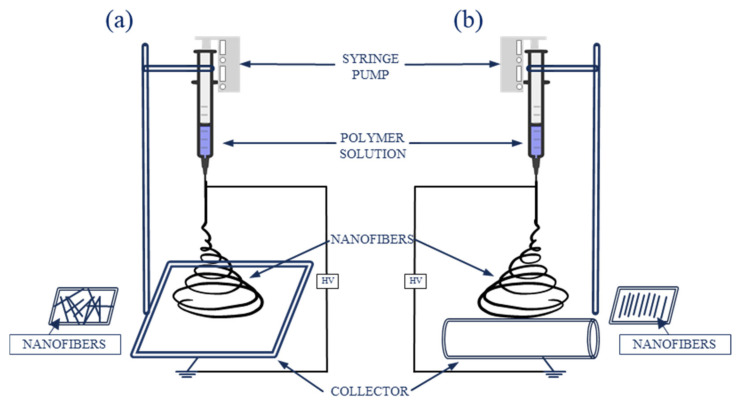
Schematic electrospinning setup: (**a**) with a plate collector; (**b**) with a rotary collector.

Standard electrospinning setup consists of a syringe filled with a solution of a polymer of choice, connected to a spinneret (for instance a needle or a pipette tip), a syringe pump, a grounded collector (either a plate or rotary), and a high voltage power supply (Figure 5). The setup can be placed vertically or horizontally (Figure 5) [24,26].

During electrospinning, an electrohydrodynamic process occurs. The polymer solution, because of the surface tension, forms a droplet at the end of the needle. When a charge is injected into a polymer solution, because of the electric field generated between the needle and the collector, the droplet forms a Taylor cone. When the electric force overcomes the surface tension, a charged jet is ejected. At first, the jet extends in a straight line, then vigorous whipping motions occur, resulting in evaporation of the solvent, while the polymer is stretched and elongated, creating solid fibers with fine diameters, deposited on the grounded collector [22,24].

Both natural (e.g., cellulose) and synthetic (e.g., polyacrylonitrile, PAN) polymers, as well as copolymers, can be used for electrospinning [24]. The parameters that determine whether the polymer can be electrospun include viscosity, conductivity, molecular weight, and solubility in proper solvents [25]. Appropriate selection of process conditions, e.g., flow rate, applied voltage, the distance between the needle and the collector, needle diameter, polymer concentration, collector type and rotation speed (in case of a rotary collector), temperature, and humidity, ensures control over the morphology of obtained nanomaterials [26,27,28].

Several electrospinning techniques have been developed, enabling the production of different nanofiber structures. For instance, the productivity of a standard process can be enhanced by multineedle or needless electrospinning, core-shell structures can be generated using a co-axial technique, where two different polymer solutions are present, and co-electrospinning allows to obtain single and bilayer nanofibers [26].

### 3.2. Nanofibers

Nanofibers represent a type of one-dimensional nanomaterials that are characterized by having one dimension significantly larger than two other dimensions [29]. Apart from their nanoscale size, NFs have attracted special attention due to many properties, including large surface area, porosity, ease of fabrication, good chemical, physical, and mechanical properties, flexibility, as well as possible control of the morphology [30,31]. Furthermore, NFs can be easily functionalized to give them additional properties necessary for use in various fields. Appropriately modified NFs have found applications in textile industry, chemistry, and environmental protection (catalysts, cells, batteries, energy storage, toxic waste removal), but also in biomedical industries, for example in wound healing, drug delivery, scaffolds engineering, sensors, and biosensors [32]. Various morphologies of NFs can be obtained, e.g., solid, porous, ribbon, core-shell, hollow, beaded, multichannel [33,34]. Carbon nanofibers (CNFs) can be obtained from electrospun polymer NFs through stabilization and carbonization. Their surface and chemical characterization often is based on microscopic (e.g., optical, scanning electron (SEM), transmission electron (TEM), atomic force microscopy (AFM)) and spectroscopic (Fourier-transform infrared spectroscopy (FT-IR), Raman, energy dispersive spectroscopy (EDS), X-ray diffraction (XRD)) techniques, respectively [35].

**Figure 6:**
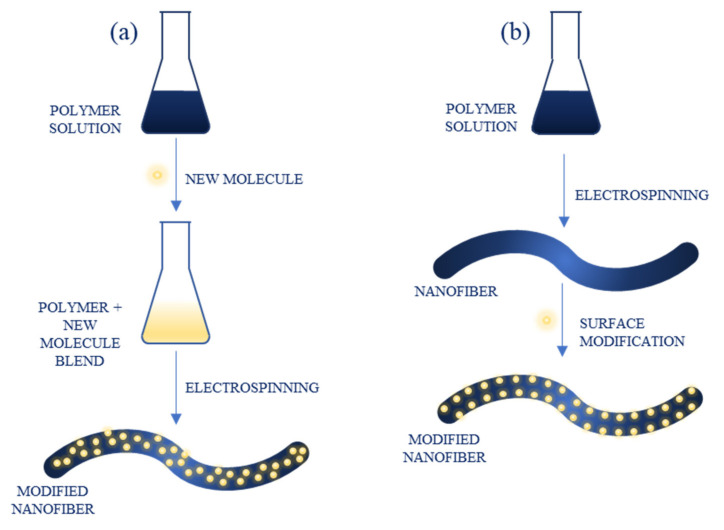
Functionalization of nanofibers through: (**a**) direct blending; (**b**) surface modification.

There are two approaches to NFs functionalization: direct incorporation, in which new compounds are introduced into the polymer solution before electrospinning, resulting in them being embedded into the structure of obtained material, and surface modification, in which new compounds are presented onto the surface of already prepared NFs (Figure 6). The molecules that can be used for NFs modification are diverse and include, e.g., polymers, nanoparticles (metallic, semiconducting-such as quantum dots), organic compounds and dyes, carbon nanomaterials and biomolecules, such as enzymes [31]. Because of a large surface area and porous structure, the capacity of biomolecule immobilization is increased compared to microscale materials, improving the performance of sensors in terms of sensitivity and response time [36].

## 4. Nanofiber-Based Electrochemical Sensors

Electrospun nanofibers have been applied in electrochemical sensors for the detection of various compounds, including metal ions, biomedically relevant molecules, and microorganisms.

### 4.1. Sensing of Glucose

One of the most popular sensors is a glucometer which monitors the level of glucose in body fluids. Several glucose sensors have been developed with the use of nanofibers. For example, Sapountzi et al. used a two-step approach-electrospinning and vapour phase polymerization-to produce core-shell nanofibers of PAN and Fe(III) p-toluenesulfonate hexahydrate (FeTos) coated with polypyrrole (PPy), using a mixture of pyrrole (Py) and pyrrole-3-carboxylic acid (Py3COOH) (1:2) as monomers [37]. Electrospun PAN NFs were deposited directly on a gold electrode and exhibited the average diameter of 677 ± 20 nm. They were impregnated with FeTos, annealed at 70 °C, and coated with a conductive layer of polypyrrole, creating PAN/PPy/PPy3COOH NFs. After this process, an enzyme (glucose oxidase, GOx) was covalently immobilized on the modified electrodes through a reaction with EDC and NHS. The electrode was characterized and tested for glucose detection using EIS method. The biosensor achieved linearity in a wide range of glucose concentrations (20 nM–2 μM), low LOD (2 nM), as well as good stability and selectivity (tested towards ascorbic and uric acids). 

Another group used a carbon nanofiber membrane decorated with NiMoO_4_ NPs for enzymeless glucose detection [38]. NFs were electrospun from a 10 wt% PAN solution in N,N-dimethylformamide (DMF) (average diameter 500 nm), stabilized, carbonized (average diameter 300 nm), and pretreated with a mixture of sulfuric and nitric acids, resulting in a carboxylated CNF membrane, on which NiMoO_4_ NPs were synthesized. The morphology and electrochemical properties of obtained membrane were characterized. Sensing ability of the NiMoO_4_/CNF was tested with CV technique, using a conventional three-electrode system (CNFs as a working electrode, Ag|AgCl as a reference electrode, and a Pt wire as an auxiliary electrode), and EIS. The results showed linearity in a wide concentration range (0.0003–4.5 mM), low LOD (50 nM), and good selectivity. In real sample analysis, the sensor exhibited a good recovery of 97.5–103.2% and the performance comparable to the results obtained by a commercial glucometer.

An electrochemical biosensor for the detection of glucose using graphene oxide (GO) nanofibers, gold nanoparticles (AuNPs), and copper nanoflowers was designed by Baek et al. [39]. Poly(vinyl alcohol) (PVA) and GO served as the electrospinning solution to obtain nanofibers with the average diameter of 168 nm directly on a gold chip, which were later decorated with AuNPs. Then copper nanoflowers with immobilized enzymes-GOx and horseradish peroxidase (HRP)-were deposited on modified NFs. The whole structure was bonded to the gold chip with Nafion as a binding agent. The presence of AuNPs improved the conductivity, sensitivity, thermal resistance, mechanical and electrical properties of the sensor. The sensor and its components were characterized with various imaging techniques (e.g., TEM, FT-IR), and the detection capabilities were tested using chronoamperometry and CV with a conventional three-electrode cell (Ag|AgCl and Pt as reference and counter electrodes, respectively). Under optimized conditions, the sensor exhibited a wide linear range of 0.001–0.1 mM and the LOD of 0.018 μM.

Ni/CoO loaded carbon nanofibers were developed by Mei group using anionic surfactant-assisted equilibrium adsorption method and electrospinning [40]. PAN was added to Ni/Co and sodium dodecyl sulfate solution and after stirring the solution was electrospun. Obtained NFs were then per-oxidized and carbonized. For electrochemical measurements, a glassy carbon electrode (GCE) modified with prepared NFs and CV technique were used. The sensor was tested for glucose determination at different concentrations, resulting in a linear range of 0.25–600 μM and LOD of 0.03 μM. Interference, stability, and real sample studies proved good selectivity and stability of constructed device. 

Fu et al. presented a non-enzymatic glucose sensor with a glassy carbon electrode modified with Ni-Co layered double hydroxide-coated CNFs (CNF@Ni-Co LDH) [41]. Pure carbon nanofibers with diameters in the range of 300–500 nm were obtained by electrospinning of PAN solution and annealing at 1000 °C. Then, chemical deposition was used to functionalize the NFs with Ni-Co LDH, resulting in a uniform flake-like structure on the surface of nanofibers. Finally, a GCE was modified with obtained nanocomposite. After the characterization of the material, its sensing performance towards glucose was tested with CV technique. Prepared sensor exhibited a low detection limit of 0.03 μM, a linear range of 1–2000 μM, and high sensitivity, good selectivity, reproducibility, and recovery.

**Figure 7:**
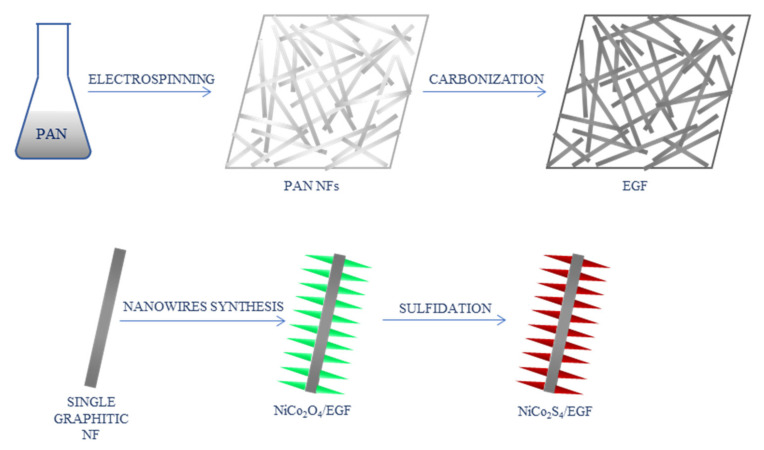
Schematic process of the synthesis of NiCo_2_S_4_-modified graphitic nanofibers [42]; PAN—polyacrylonitrile, NFs—nanofibers, EGF—electrospun graphitic nanofiber film.

In a different study, nickel cobalt sulfide-modified electrospun graphitic nanofiber film (EGF) was used for non-enzymatic glucose biosensing [42]. Electrospinning of PAN solution and carbonization at 2000 °C were used to prepare graphite NFs. NiCo_2_S_4_ in a form of nanowire arrays were grown on graphite NFs by a two-pot hydrothermal reaction: hydrothermal synthesis of NiCo_2_O_4_ nanowires and chemical sulfidation (Figure 7). The composite was used to modify a GCE. CV was applied for electrochemical measurements. The LOD of 0.167 μM and a wide linear range of 0.0005–3.571 mM were obtained. In addition, the sensor presented good sensitivity and selectivity, stability, and excellent reproducibility.

Adabi and Adabi also developed a non-enzymatic glucose sensor with the use of nickel-carbon NFs [43]. CNFs were prepared from PAN solution via electrospinning, stabilization, and carbonization. Electrodeposition was used to place nickel particles on the surface of CNFs. Obtained nanofibers were characterized and used as a working electrode for glucose sensing, with Ag|AgCl reference electrode and Pt wire as an auxiliary electrode. The average diameter of CNFs was equal to 85.3 nm, which was a reduced result compared to the initial PAN NFs (124.4 nm). The presence of Ni on CNFs was confirmed by SEM, EDS, and XRD. The results of the electrochemical measurements presented the LOD of 0.57 μM, a linear range of 2 μM–5 mM, good selectivity and stability, as well as short response time (2 s). The sensor was also tested on real samples, proving to be promising for the detection of glucose in clinical applications.

Carbohydrate polymers can serve as a base for the nanofibers. Yezer and Demirkol blended cellulose acetate (CA) with chitosan (CS) to obtain electrospun NFs [44]. After optimization, smooth NFs were collected directly on the GCE. Depending on the CA concentration, diameters of the NFs varied between 40 and 355 nm. After drying, glucose oxidase was immobilized on the modified electrode with glutaraldehyde as a cross-linking agent. The enzyme activity was tested, and the electrode was characterized electrochemically using CV, DPV, and EIS. Performed experiments revealed the detection limit of 4.8 μM and the linear range of 5.0 μM–0.75 mM. Tested interfering agents did not affect the performance of the sensor. Real sample analysis was also carried out, showing recovery between 96.5% and 103.0% (depending on the type of tested sample: artificial tears, urine, sweat, and serum).

**Figure 8:**
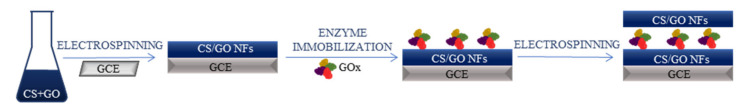
Schematic process of the synthesis of GOx/CS/GO nanofibers [45]; CS—chitosan, GO—graphene oxide, GCE—glassy carbon electrode, GOx—glucose oxidase.

Mehdizadeh et al. also used chitosan to prepare glucose sensor-CS/GO nanofibers, with the enzyme trapped between two NFs layers, which were used to modify a GCE [45]. The NFs were electrospun directly on the electrode surface. The average diameter was between 105.11 and 386.49 nm, depending on the GO concentration. Then, the enzyme was immobilized on the nanofibers and after drying, another layer of NFs was deposited on the first layer to ensure that the enzyme stayed on the NFs (Figure 8). Obtained nanostructures were analyzed using, i.a., microscopy, XRD, and FT-IR. Electrochemical studies were performed using CV with a conventional three-electrode system (modified GCE, Ag|AgCl and a Pt wire served as working, reference, and counter electrodes, respectively). The sensor presented linearity in a range of 0.05–20 mM, good selectivity and stability, and the detection limit was found at 0.02 mM.

Another glucose biosensor based on GOx and NFs was designed by Temoçin [46]. An H_2_O_2_-sensitive GCE was modified with electrospun poly(ethyleneimine) (PEI)/PVA NFs (diameter 350–500 nm). The enzyme was immobilized on the surface of GCE with glutaraldehyde. UV-vis and electrochemical measurements were performed on the obtained electrode, and the NFs were characterized by SEM and FT-IR. The LOD of the biosensor, found based on DPV measurements, was 0.3 mM and a linear range was found at 2–8 mM and 10–30 mM. Additional tests confirmed the selectivity and stability of the sensor, and real sample analysis was performed with satisfying results.

Nanofiber-based sensor systems for the detection of glucose are summarized in Table 1.

### 4.2. Sensing of Biomedically Relevant Molecules and Drugs

Electrochemical sensors based on nanofibers were developed towards various biomolecules and drugs, for instance, neurotransmitters, hormones, enzymes, and antibiotics.

Ratlam et al. presented a biosensor based on polyaniline (PANi)/carbon QDs composite (CQDs) for the detection of a neurotransmitter-dopamine [49]. Electrospinning technique was used to obtain a nanofibrous matrix with the average diameter of NFs equal to 320 nm, and various techniques were used for its characterization, including SEM and FT-IR. For the electrochemical measurements, CV was applied, using a conventional three-electrode system, with NFs on fluorine doped tin oxide-coated glass substrate as a working electrode, Ag|AgCl as a reference electrode and a Pt wire as a counter electrode. The biosensor exhibited a low LOD of 0.1013 μM and a linear range of 10–90 μM, as well as good sensitivity and selectivity.

Ozoemena et al. used onion-like carbon and its carbon nanofiber composite (OLC-CNF) for dopamine detection [50]. PAN and OLC served as precursors for electrospinning, and obtained NFs were later stabilized and carbonized, resulting in OLC-CNF (diameter of NFs 475–800 nm). The nanostructure was characterized and used to modify GCE, which served as a working electrode, alongside Ag|AgCl and Pt as reference and counter electrodes, respectively. The electrochemical characterization was based on CV, EIS, and square wave voltammetry. These tests allowed to find the LOD at 1.42 μM. Further examination showed selectivity, sensitivity, and applicability in real samples (tested for dopamine detection in its pharmaceutical formulation).

Nathani and Sharma presented a biosensing performance of mesoporous poly(styrene-block-methyl-methacrylate) (PS-b-PMMA) nanofibers towards streptavidin as a model analyte [51]. The authors also explored how the sensing ability was influenced by the porosity of NFs. The block copolymer was electrospun into NFs with the average diameter of 336 ± 73 nm, which were later used to functionalize GCE. The NFs were further functionalized with biotin-hydrazine solution via EDC/NHS linking and blocked with bovine serum albumin (BSA). CV and DPV methods were used for electrochemical studies. The results indicated selectivity of the sensor, with the LOD for streptavidin at 0.37 fg/mL and a wide detection range of 10 fg/mL–10 ng/mL.

The use of cellulose acetate NFs allowed the detection of 25-hydroxy vitamin D_3_ [52]. CA NFs were electrospun onto a conducting paper substrate, which served as an electrode. NFs characterization showed the average diameter of 330 ± 3.5 nm. For vitamin D_3_ detection, a specific monoclonal antibody was immobilized and blocked on the electrode and a chronoamperometric study was conducted. Obtained immunosensor showed selectivity and sensitivity, a wide linear range of 10–100 ng/mL, and a low LOD of 10 ng/mL. The results were validated using ELISA technique of serum samples.

Hybrid nanofibers composed of cellulose monoacetate and Nafion (CMA/N) were used in a DNA biosensor [53]. The CMA/N blend was electrospun, resulting in NFs with the average diameter between 500 nm and 1.5 μm, which were analyzed using methods such as SEM and FT-IR. The sensing performance was focused on the oxidation of guanine of immobilized DNA. Pencil graphite electrodes were functionalized with NFs and single strand DNA probes, unmodified and NH-modified. DPV measurements were performed in a three-electrode system, with Ag|AgCl and a Pt wire as reference and counter electrodes, respectively. The oxidation signals were compared for both types of DNA and NFs with different CMA to Nafion ratio, reaching maximum for pure CMA NFs and unmodified DNA.

Wang et al. synthesized MoS_2_ nanosheet arrays/carbon nanofibers (MoS_2_ NSA/CNF) and employed them in a sensor for simultaneous detection of levodopa, a dopamine precursor, and uric acid [54]. The composite was obtained using electrospinning of PAN to synthesize CNFs with the average diameter of 200 nm, and hydrothermal synthesis to grow NSA on the surface of NFs, and it was characterized with SEM and XRD. For the electrochemical measurements, CV and DPV methods were used. The results showed the LOD of 1 μM and a linear range of 1–60 μM for both analytes, as well as good sensitivity, selectivity, reproducibility, and stability of the electrode.

In another work, honey/PVA core-shell nanofibers, multi-walled carbon nanotubes (MWCNTs), and AuNPs, as well as an aptamer, were used to modify a GCE for the impedimetric determination of MUC1, a breast cancer marker [55]. Co-axial electrospinning was used to obtain NFs, collected directly on the electrode, with the average diameter of 100 ± 15 nm. Then, the electrode was functionalized with AuNPs and MWCNTs. The aptamer was immobilized after activating the carboxylic groups of the nanotubes via EDC/NHS reaction. SEM and FT-IR were used to characterize obtained material, and the electrochemical measurements were carried out using EIS and CV methods. The sensor showed a wide linear range of 5–115 nM and the detection limit equal to 2.7 nM. Further tests revealed good stability, sensitivity, and selectivity, and satisfactory real sample analysis results.

Nanofibers modified with AuNPs and MWCNTs for breast cancer biomarker determination was also investigated by Adabi et al. [56]. In this study, CNF mat functionalized with AuNPs, cysteamine, MWCNTs, and antibody, was used for the detection of human epidermal growth factor receptor 2 (Her-2). The characterization of obtained electrode was based on field emission SEM (FESEM) and EDS, and CV was adopted for the electrochemical performance examination. The immunosensor reached a low LOD of 0.45 ng/mL and a linear range of 5–80 ng/mL. Good reproducibility and repeatability were obtained. The performance of the sensor was confirmed by real sample analysis.

Nicotine was the target of a sensor based on MWCNT/CS NFs [57]. Functionalized MWCNTs and chitosan blend was used for the electrospinning, resulting in NFs with the average diameter between 95.47 nm and 131.01 nm, depending on the MWCNTs percentage. Obtained nanostructure was collected directly on the GCE. Its characterization included FESEM, TEM, AFM, and FT-IR. For the electrochemical measurements, CV was used. The results showed a linear range between 0.1 and 100 μM and the detection limit of 30 nM. The sensor exhibited good selectivity, sensitivity, stability, and reproducibility.

The NiO-Au hybrid NFs were adopted in an aptasensor for the determination of progesterone (P4), a steroid hormone [58]. The NFs were electrospun from a composite consisting of Ni(NO_3_)_2_, HAuCl_4_, and polyvinylpyrrolidone (PVP), and were then functionalized with graphene QDs (GQDs). A screen printed carbon electrode was used as a working electrode after modification with GQDs-NiO-Au NFs and MWCNTs. The P4-specific aptamer was immobilized on the electrode via EDC/NHS reaction and blocked with BSA. For electrochemical experiments CV, EIS, and DPV techniques were used. The aptasensor showed great selectivity and stability, with the LOD of 1.86 pM and a linear range of 0.01–1000 nM.

Nanofiber-based sensor systems for the detection of biomolecules are summarized in Table 2.

Several publications concerning NF-based sensors toward drugs have been reported. For example, Vafaye et al. presented a study of an AuNP/CNF aptasensor for the determination of penicillin in milk samples [66]. CNFs were obtained by electrospinning from a PAN solution and carbonization. The electrodeposition was adopted to functionalize NFs mat with AuNPs. Lastly, a penicillin aptamer was immobilized on the surface of the nanostructure. SEM, EDS, and Raman spectroscopy were used for the characterization of the electrode, and the electrochemical measurements were conducted with the CV method, with Ag|AgCl as a reference electrode and a Pt wire as an auxiliary electrode. The results indicated good selectivity, stability, and reproducibility of the sensor, a wide linear range of 1–400 ng/mL, and the LOD was found at 0.6 ng/mL. The sensor was validated by comparison with HPLC results.

Modified CNFs were also used in a sensor for colchicine detection [67]. The authors synthesized CuO NPs and added them to a PVA solution, which was later used for the fabrication of NFs through electrospinning. Obtained NFs were then stabilized and carbonized to give CuO/CNFs. A magnetic ionic liquid (MIL), 1-butyl-3-methylimidazolium tetrachloroferrate ([Bmim]FeCl_4_) was also prepared. GCE was modified with both CuO/CNF and MIL, and Nafion was cast on the surface for binding. Obtained materials were characterized (e.g., SEM, FT-IR), and CV and DPV methods were used for electrochemical measurements. The average diameter of the spherical CuO NPs and CNFs was 48 nm and 80 nm, respectively. The linear range and the detection limit of the sensor were 1–100 nM and 0.25 nM, respectively. In addition, the sensor exhibited good sensitivity, selectivity, and stability and was used for real sample analysis with satisfactory results.

Acetaminophen (APAP) and p-hydroxyacetophenone (p-HAP) were simultaneously detected by a sensor utilizing a composite SnO_2_-CNF [68]. PAN mixed with SnCl_2_·2H_2_O served as a precursor for the NFs, which were later carbonized to obtain SnO_2_-CNF with a diameter in the range of 400–500 nm. This material was used to modify a GCE. Microscopic and spectroscopic techniques were used for the characterization of the electrode, and EIS and DPV methods were adopted for electrochemical measurements. The sensor showed great reproducibility, repeatability, and stability, as well as high selectivity proven by the interference study. It was also successfully applied in real sample analysis. For APAP, the linear range was 0.5–700 μM with the LOD of 0.086 μM, while for the p-HAP these parameters were 0.2–50 μM and 0.033 μM, respectively.

Bahrami et al. developed a sensor based on magnetic nanofibers for the detection of morphine [69]. The NFs were prepared from PVA and iron salt precursors via electrospinning and a thermal process and had diameters in a range of 70–120 nm. Graphite powder, paraffin oil, and NFs were used to obtain a modified carbon paste electrode (CPE). Electrochemical studies were performed using CV and DPV techniques. XRD, SEM, and FT-IR were used for the characterization of the electrode. The sensor exhibited good sensitivity and selectivity, with a detection limit of 1.9 nM and a wide linear range of 0.0033–245 μM.

Table 3 summarizes NFs-based sensors for the electrochemical detection of drugs.

**Figure 9:**
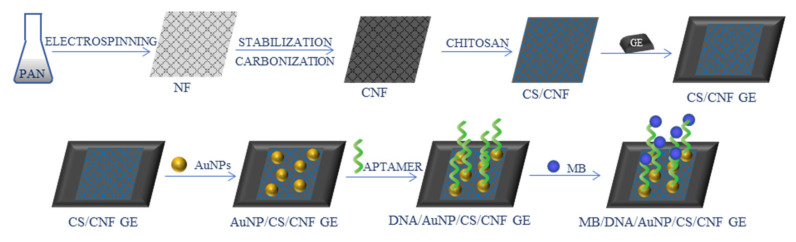
Schematic process of the synthesis of Salmonella enterica aptasensor [73]; PAN—polyacrylonitrile, NF—nanofibers, CNF—carbon nanofibers, CS—chitosan, GE—graphite electrode, AuNPs—gold nanoparticles, MB—methylene blue.

An aptasensor for *Salmonella enterica serovar* was developed by Fathi et al. [73]. CNFs (average diameter 90 ± 10 nm) were fabricated from PAN precursor, mixed with CS, and deposited on a pencil graphite electrode. The surface of the electrode was further modified with AuNPs, an aptamer, and methylene blue (MB) (Figure 9). The obtained sensor was electrochemically characterized by CV and EIS techniques. The LOD was found at 1.223 CFU/mL, and the sensor showed a wide linear range of 10–10^5^ CFU/mL, good selectivity, reproducibility, and stability.

Liberato et al. presented an immunosensor for the detection of *Leischmania braziliensis*, where the recognition was based on an interaction between an epitope designed from the antigen and its specific antibody [74]. Polyamide 6 (PA6)/CS NFs were fabricated using the electrospinning technique (diameter in a range of 15–170 nm) and characterized with, i.a., SEM, XRD, and FT-IR. For electrochemical studies, CV and EIS were adopted. Cellulose acetate covered by a gold layer was used as a working electrode. The electrode was modified with NFs, promastigote surface antigen, and BSA. The results showed a low detection limit of 1.6 pg/mL and a linear range of 2.5–10 pg/mL (of specific antibodies). The sensor was also examined by real sample analysis that included both positive and negative serum samples.

In a different work, functionalized CNFs were used for the detection of *Pseudomonas aeruginosa* [75]. The authors prepared hollow carbon nanocapsules-based nitrogen-doped carbon nanofibers (CNCNF) with a rosary-like structure. To do that, Fe_2_O_3_ nanocapsule templates were covered by a layer of polydopamine (which served as a carbon source), were later transformed into carbon N-doped nanocapsules and the iron oxide core was etched. NFs were fabricated from a PAN and nanocapsules solution via electrospinning and carbonization. Obtained CNCNF were used for the modification of a GCE. In order to immobilize the aptamer on the electrode surface, it was covered with a layer of mercaptopropionic acid, and EDC/NHS coupling was used to secure the biomolecule on the electrode. The electrochemical measurements were performed using CV and EIS methods. The sensor presented good stability, selectivity, repeatability, satisfactory results in real sample analysis, with a linear range of 10–10^7^ CFU/mL and the LOD at 1 CFU/mL.

Electrospun CNFs were also applied in a sensor for a hepatitis B virus detection [76]. CNFs were prepared from a PAN solution (diameter between 70–282 nm, depending on DMF/acetone ratio) and examined with SEM, XRD, and Raman spectroscopy, and served as the electrode. Electropolymerization was used to modify the CNF electrode with glutamic acid, to which a DNA fragment was attached via EDC/NHS reaction to fabricate a biosensor. CV was adopted for electrochemical measurements, which revealed a detection limit equal to 1.58 pM and a wide linear range of 1 pM–1 μM, as well as good selectivity and stability.

### 4.3. Sensing of Metal Ions

Electrospun NFs have also found application in the detection of metal ions. For example, Ehzari et al. constructed an aptasensor for Hg^2+^ detection using CPE modified with polyethersulfone NFs, with the average diameter of 130 nm, and thiol-capped CdTe QDs [77]. In this work, nanomaterials served as an amplifier for the signal generated by an interaction between mercury ions, thymine, and methylene blue. NFs-QDs nanocomposite was collected directly on the CPE during the electrospinning process. The surface of the electrode was activated with EDC/NHS to immobilize an aptamer, and it was subsequently immersed in MB. The sensor was then exposed to various Hg^2+^ solutions, then immersed in MB again. Characterization of the sensor and its components was performed (e.g., SEM, FT-IR, EDX). The electrochemical sensing performance was examined with DPV. The results showed good specificity, stability, and repeatability of the sensor, with a wide linear range of 0.1–150 nM and the detection limit of 0.02 nM.

Another aptamer sensor for mercury ions detection was developed by Xie et al. [78]. Carbon ionic liquid electrode was functionalized with platinum nanoparticles (PtNPs)/CNF and AuNPs. The NFs were fabricated from a PAN solution through electrospinning and carbonization and had the average diameter of 400 nm. Then, PtNPs were formed on the CNF. This composite was used to modify the electrode, and then AuNPs were synthesized on the surface and the aptamer was immobilized on the electrode. As in the previous example, the sensing mechanism was based on the Hg^2+^-thymine binding. Electrochemical measurements were conducted using CV and EIS techniques, showing a wide linear range (1 fM–1 μM) and a low LOD of 0.33 fM. Selectivity, stability, and repeatability were also confirmed by the test, and real sample analysis gave satisfactory results.

A different approach to Hg^2+^ detection was presented by Teodoro et al. [79,80]. They used a nanocomposite composed of PA6 NFs (average diameter of 130 ± 32 nm), on which carbon nanowhiskers and reduced GO hybrid material was adsorbed. After characterization, electrochemical measurements were performed, using DPV and CV techniques, in a conventional three-electrode system, with fluorine tin-oxide electrode modified with the obtained nanocomposite as a working electrode, Ag|AgCl as a reference electrode, and Pt as a counter electrode. The LOD and the linear range were found at 0.52 μM and 2.5–200 μM, respectively. An interference study confirmed selectivity, and the sensor was tested on real samples.

Liu and Zhang used nitrogen-doped CNF with metal-organic NPs for Cd^2+^ and Pb^2+^ ions detection [81]. ZIF-8 NPs were prepared and added to PAN solution, from which NFs were electrospun and carbonized in a nitrogen atmosphere, resulting in nitrogen-doped CNFs. GCE modified with CNFs was used as a working electrode. For the electrochemical measurements, anodic stripping voltammetry and differential pulse anodic stripping voltammetry were used. The sensor showed a linear range of 2–100 μg/L and 1–100 μg/L, and the LOD of 1.11 μg/L and 0.72 μg/L for Cd^2+^ and Pb^2+^, respectively. The selectivity was confirmed by an interference study, and real sample examination showed acceptable results.

A Pb^2+^ sensor was also presented by Oliveira et al. [82]. This sensor was based on L-cysteine modified ZnO NFs (average diameter of 335 nm). The NFs were fabricated from a PVA-zinc acetate solution via electrospinning and annealing, and then functionalized with L-Cys. This composite was used to modify a GCE. The NFs and the electrode were characterized, i.a., with SEM and FT-IR. Electrochemical measurements were carried out using square wave anodic stripping voltammetry (SWASV). The results indicated good stability, selectivity, and repeatability, and the LOD and the linear range were 0.397 μg/L and 10–140 μg/L, respectively.

Modified CNFs were adopted for As^3+^ determination [83]. Fe-CNFs were prepared from a PAN and iron acetylacetonate solution, then polyaniline was polymerized on their surface and finally, the composite was decorated with AuNPs. The obtained material was used for a GCE modification. SEM, TEM, XRD, and X-ray photoelectron spectroscopy (XPS) techniques were used for the characterization, and CV and SWASV were used for the electrochemical analysis. Excellent sensitivity and selectivity were achieved, with a low LOD at 6.67 nM in a linear range of 0.07–5.34 μM.

Sensors for the detection of metal ions are summarized in Table 4.

## 5. Nanofiber-Based Optical Sensors

Next to electrochemical sensors, optical devices are often used for the detection of various compounds.

### 5.1. Sensing of Metal Ions

**Figure 10:**
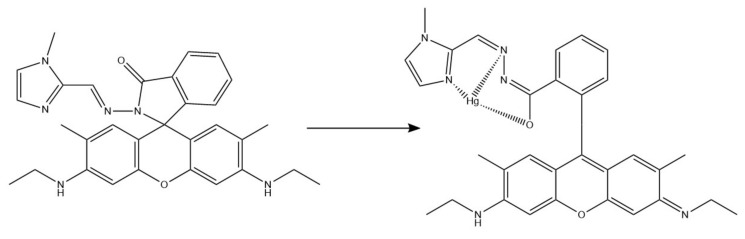
Possible binding mechanism between RIM and Hg^2+^ [84].

For optical mercury ions sensing, Rao et al. developed a colorimetric and turn-on fluorescence chemosensor based on rhodamine 6G [84]. They synthesized a rhodamine derivative functionalized with N-methyl imidazole unit (RIM), which was added to polyurethane (PU) solution to obtain nanofibers. The sensing performance was tested with UV-Vis and fluorescence emission, and ^1^H NMR and electrospray ionization mass spectrometry methods were used to understand the binding mechanism between Hg^2+^ and RIM (Figure 10). In both day and UV light, an instant color change (from colorless to pink and fluorescent yellow, respectively) was noticeable in the solution containing RIM and Hg^2+^ ions, with no change in case of different ions, proving that the sensor was selective. Similarly, Hg^2+^ was the only tested metal ion (out of 16) that produced an additional peak on absorption and fluorescence emission spectra (at 538 nm and 562 nm, respectively). The use of NFs allowed a rapid, colorimetric, on-site detection of mercury ions, as prepared RIM-NFs strips changed the color to pink under UV light upon Hg^2+^ addition.

**Figure 11:**
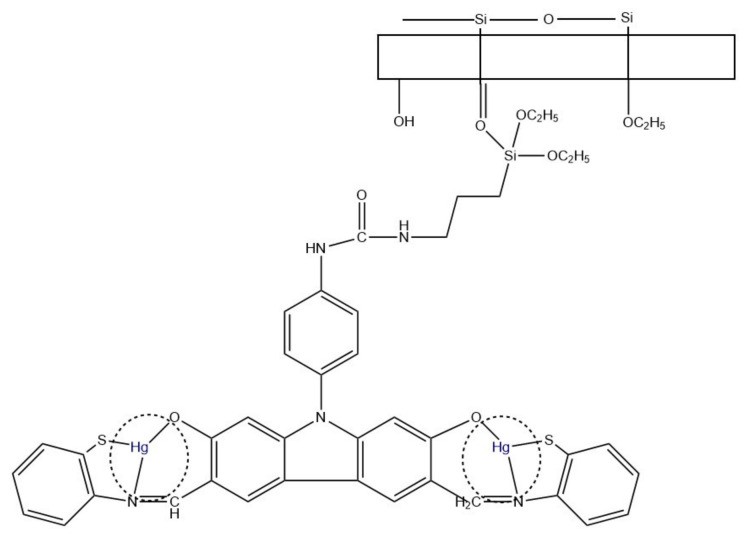
Hg^2+^ removal mechanism using PVA/TEOS/S membrane [85].

A different Hg^2+^ sensor was developed by Tahvili et al. [85]. In this study, the detection and removal of mercury ions was possible thanks to a carbazole-based Schiff base (S) immobilized on a PVA-tetraethyl orthosilicate (TEOS) polymer (Figure 11). The whole PVA/TEOS/S structure was fabricated via electrospinning, and the diameter varied between 57–110 nm, depending on the ratio. NFs and the Schiff base were characterized using, i.a., FT-IR and FESEM. Performed experiments showed the efficiency of Hg^2+^ removal from aqueous solutions of 98.04–94.26%. The interference study included 12 different metal ions and proved the selectivity of the sensor. The LOD equal to 0.018 ng/mL and a linear range of 0.02–0.5 ng/mL were found.

**Figure 12:**
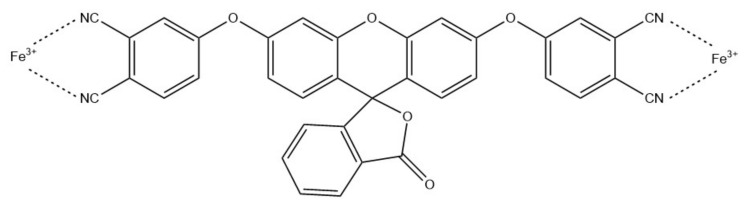
Schematic Fe^3+^ chelation of FPN [86].

Iron (III) ions can also be detected using NFs-based optical sensors. For example, Rijin et al. functionalized polycaprolactone (PCL) NFs with a 4,4′-fluoresceinoxy bisphthalonitrile (FPN) fluorophore to create a membrane strip capable of selective determination of Fe^3+^ (Figure 12) [86]. NFs were prepared via electrospinning and characterized using, e.g., SEM and FT-IR. Their average diameter was in the range of 350–500 nm. The selectivity and sensitivity studies, based on fluorescence measurements, showed significant fluorescence quenching upon adding Fe^3+^ ions with the LOD at 2.94 nM.

Zhang et al. used poly(aspartic acid) (PASP) NFs for a colorimetric sensor for the determination of Fe^3+^ and Cu^2+^ [87]. To do that, polysuccinimide NFs were electrospun, cross-linked and hydrolyzed, resulting in poly(aspartic acid) electrospun nanofiber hydrogel membrane. A large surface area of PASP NFs and its ability to adsorb Cu^2+^ ions allowed a colorimetric detection of these ions through color change to blue. In the case of Fe^3+^, because of a smaller adsorption ability, filtration was required for the membrane to capture iron (III) ions. In this case, the color changed to yellow. The detection limits and color changes, based on naked eye observations, were found at 0.3 mg/L and a range of 0.3–30 mg/L for Cu^2+^, and 0.1 mg/L and a range of 0.1–10 mg/L for Fe^3+^, respectively.

**Figure 13:**
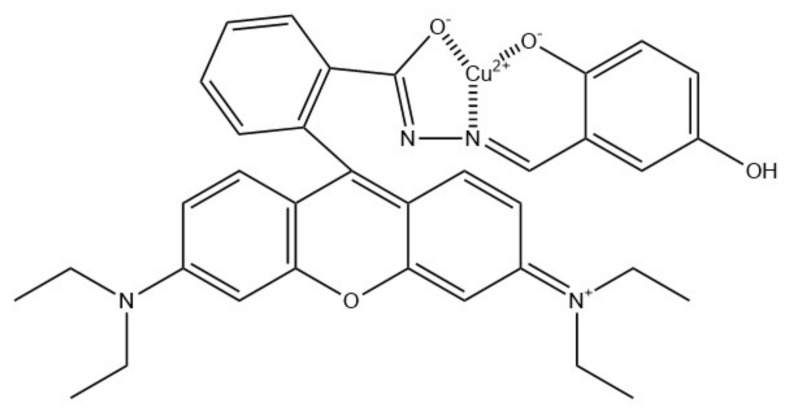
Proposed binding mechanism between rhodamine B derivative and copper(II) ions [88].

A colorimetric Cu^2+^ sensor was also developed by Tungsombatvisit et al. [88]. This work was based on rhodamine B derivative (RBD) cellulose acetate NFs (average diameter of 750 ± 270 nm), with RBD turning pink in contact with Cu^2+^ (Figure 13). To obtain them, RBD and CA solutions were mixed and electrospun, and later alkaline treatment by immersion in NaOH was applied in order to deacetylate the CA. Sensing performance evaluation was based on a color change of the NFs, examined with a spectrophotometer in terms of color strength and color values (CIELab coordinates). Performed experiments revealed the LOD of 0.28 mM and a linear range of 0.3–4.7 mM.

Another Cu^2+^ optical sensor was based on NFs and QDs [89]. The authors used two types of QDs–green and red emitting (QDs_g_ and QDs_r_, respectively). QDs_r_ were functionalized with PEI via ligand exchange, while a solution of QDs_g_ and poly(vinylidene fluoride) (PVDF) was electrospun into NFs with the average diameter of 300 nm. Both composites were then shaken together to produce a dual fluorescent film through adhesion, where QDs_r_/PEI covered QDs_g_/PVDF NFs, allowing the use of 365 nm UV light for the emission of two colors. Quenching of the QDs fluorescence occurred upon contact with Cu^2+^. However, QDs_g_ were protected by polymer NFs and therefore their fluorescence did not significantly change under these conditions, in contrast to QDs_r_ fluorescence. This sensor showed the visual LOD at 2 μM, short response time (30 s), good selectivity and stability. It was also tested on real water samples with satisfactory results.

The same group developed another QDs/NFs based Cu^2+^ sensor [90]. In this work, CdSe/Cd_x_Zn_1-x_S QDs were synthesized and deposited on the surface of electrospun PA6 NFs (average diameter of 68 nm) via dip-coating. As previously, 365 nm UV light was used to observe the fluorescence after Cu^2+^ addition. Morphology and optical properties of obtained materials were characterized using TEM, SEM, and spectrophotometer measurements. The application of nanofibrous matrix reduced aggregation, and therefore fluorescence quenching, making the sensor stable and more accurate. Selectivity was tested with various metal ions, with no change in the fluorescence intensity. Visual LOD was found at 10 μM with the response time of 10 s, and stability in a wide range of conditions was described.

Optical sensors for metal ions sensing are summarized in Table 5.

### 5.2. Sensing of Biomedically Relevant Compounds

Li et al. presented a sensor for one-step thrombin detection, using electrospun polystyrene NFs with the average diameter of 350 nm [91]. NFs were functionalized in three steps: firstly, they were subjected to plasma treatment, then incubated with avidin, and finally incubated in Tris buffer containing an oligonucleotide (B-H2), thus creating the sensing platform. For the fluorescence measurements, the excitation wavelength was 450 nm, and the fluorescence intensity at 492 nm was taken under consideration for sensing performance evaluation. The detection process was based on proximity-induced DNA strand displacement, catalytic hairpin assembly amplification, and thioflavin T binding. The sensor showed the LOD at 1 pM and a wide linear range of 50 pM–5 nM, good specificity, sensitivity, and stability.

A composite consisting of PANi and carbon QDs was fabricated via electrospinning and used as a dopamine biosensor [49]. The average diameter was 320 nm. Characterization methods for obtained materials included dynamic light scattering (DLS), XRD, and FT-IR. The excitation wavelength used for fluorescence measurements was 360 nm, with maximum emission at 442 nm. Conducted experiments, based on fluorescence quenching, revealed the detection limit of 0.0801 μM and a linear range of 0.1–100 μM. 

Rostami et al. also developed a dopamine sensor [92]. This platform was based on electrospun NaOH-treated poly(ethylene terephthalate) (PET) NFs (average diameter equal to 680 nm) decorated with AuNPs. Obtained materials were characterized using, i.a., SEM, DLS, and FT-IR. The sensor allowed colorimetric detection of dopamine (change from pink to navy blue) with good specificity and selectivity, despite previous reports showing a color change upon ascorbic acid addition. The linear range and LOD were found at 0.5–500 μM and 0.5 μM, respectively.

Pesticide residues can be detected by NF-based sensors, as presented by Zhai et al. [93]. Two nanofibrous mats were developed-enzyme-based (EFM) and substrate-based (SFM). For both types, PVA mixed with an accurate compound-acetylcholinesterase (AChE) or indolyl acetate (IA), respectively-was used for the electrospinning of the NFs. The average diameter of NFs was 240 ± 53 nm for EFM and 387 ± 84 nm for SFM. Characterization included SEM, FT-IR, and XPS. For the detection, the sample was added dropwise on the EFM and incubated, and then both mats were combined and incubated again, which led to an observable color change of the EFM. The LOD for different pesticides were found between 0.02 mg/L and 1.5 mg/L, the sensor also showed good stability over time.

**Figure 14:**
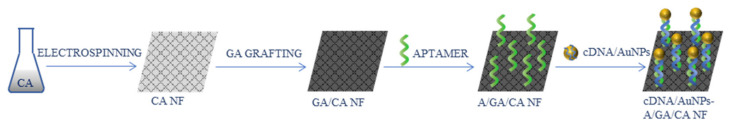
Schematic process of the synthesis of the kanamycin aptasensor [94]; CA—cellulose acetate, GA—glutamic acid, A—aptamer, cDNA—complementary single-stranded DNA, AuNPs—gold nanoparticles.

Cellulose acetate NFs-based aptasensor for a colorimetric determination of an antibiotic, kanamycin, was reported by Abedalwafa et al. [94]. The signal probe consisted of complementary single-stranded DNA (cDNA) conjugated with AuNPs. For the nanofibrous mat preparation, CA solution was electrospun into NFs, then glutamic acid (GA) was grafted onto them, forming covalent bonds between CA and GA. An aptamer was attached to functionalized NFs after EDC/NHS activation, which allowed for the formation of a hybrid with the aptamer DNA (Figure 14). The average diameter of electrospun NFs was 277 ± 65 nm. However, they showed swelling upon GA addition, increasing in diameter to 307 ± 74 nm. Both the probe and the sensing platform were characterized, e.g., using TEM, FT-IR, and XPS. The detection was based on higher affinity of the aptamer toward the analyte than towards the complementary DNA, which led to kanamycin replacing the cDNA and, as a result, to a decrease in observed absorbance intensity at 510 nm, as well as a visible color change from pink to white. Further examination confirmed good sensitivity and selectivity, low LOD (2.5 nM), and satisfactory real sample analysis.

Lee et al. adopted NFs for a colorimetric and fluorometric biothiol detection [95]. Mercury-complexed, pyridine-containing polydiacetylene (DA-Hg-DA) and polyethyleneoxide (PEO) solution was used for the electrospinning of the NFs, which were later dried and subjected to photopolymerization. Tested thiols (alkyl-, aryl-, and biothiols) caused a color change of the sensing strip from blue to red, while amines did not affect the color. The color change resulted from a decomposition of the pyridine-mercury complex. From naturally occurring amino acids, only cysteine led to a color change, and it also gave the most sensitive response of the three tested biothiols (cysteine, homocysteine, and glutathione). The use of PEO NFs for the incorporation of poly(DA-Hg-Da) allowed to increase the sensitivity of the sensor.

Zhang et al. presented a reusable sensor for a dual detection of a single droplet in a form of an indicator paper [96]. NFs were formed via electrospinning from a solution consisting of polystyrene, upconversion nanorods, and Ag@SiO_2_, and characterized, e.g., by TEM, SEM, and XRD. They had the average diameter of 200 nm. Riboflavin and pH were detected in a single droplet using a 980 nm laser at a 45° angle, and photoluminescence spectra were collected. For pH value determination, a droplet containing fluoresceine isothiocyanate (FITC) was added first. Sensing was based on the fluorescence resonance energy transfer (FRET) mechanism. The sensor exhibited excellent sensitivity, with the LOD at 0.01 ppm for riboflavin, as well as recyclability.

**Figure 15:**
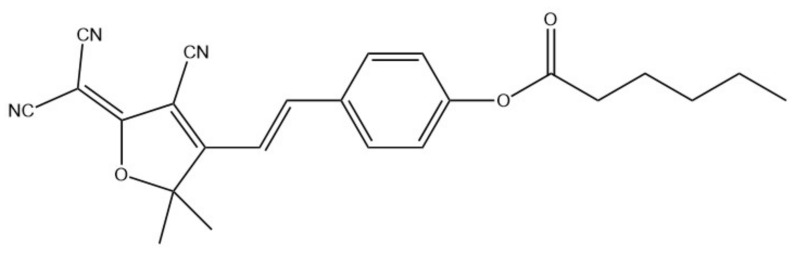
Chemical structure of HCy [97].

Core-shell electrospun NFs were used for bacterial infections detection [97]. Various NFs were electrospun, with polyurethane core and different ratios of PU/PVP and PU/polyethylene glycol (PEG) as shell, with hemicyanine based chromogenic probe (HCy, Figure 15) incorporated in the shell of the NFs. HCy and NFs were characterized using, i.a., FT-IR, TEM, and SEM. The average diameter varied depending on the composition between 150 and 249 nm. The sensor was tested on methicillin-resistant *Staphylococcus aureus* (MRSA) and *Pseudomonas aeruginosa* bacteria. Since the sensing membrane was placed directly on bacterial lawns, the colorimetric response was examined by naked eye and by measuring the reflectance spectra through the Petri dish glass. For ex vivo colorimetric response evaluation, a porcine model and a multispecies model were adopted. The change of color of the membrane from yellow to green was caused by lipase activity. Conducted experiments showed that the use of core-shell structure improved the performance of the sensor and enabled faster detection. The most optimal parameters allowed the detection of 10^5^ CFU/cm^2^ of *P. aeruginosa* and 10^6^ CFU/cm^2^ of MRSA, which are clinically relevant concentrations.

Optical sensors for various molecules sensing are summarized in Table 6.

## 6. Conclusions

The need for the specific and fast detection of various compounds in a given sample has led to the development of sensing and biosensing platforms. With the progress of science, the requirements for such platforms have changed, as more sensitive and selective determination techniques have become necessary. The incorporation of nanomaterials allowed to meet these expectations, resulting in sensing devices with higher loading capacity, faster response time, better parameters, and therefore better performance. Nanofibers, thanks to their properties such as large surface area to volume ratio, ease of functionalization, and easy fabrication, have attracted special attention in (bio)sensor design. Nanofiber-based sensors and biosensors, both electrochemical and optical, can be used to detect a variety of species, from heavy metal ions, through small molecules, to microorganisms. Because of that, they find real-life applications, for instance, in clinical diagnostics and environmental protection.

## Figures and Tables

**Table 1:** Nanofiber-based sensors for electrochemical glucose detection.

Sensor	Linear Range	LOD	Ref.
PAN/PPy/PPy3COOH	20 nM–2 μM	2 nM	[37]
NiMoO_4_/CNF	0.0003–4.5 mM	50 nM	[38]
Cu-nanoflower@AuNPs-GO NFs	0.001–0.1 mM	0.018 μM	[39]
Ni/CoO CNF	0.25–600 μM	0.03 μM	[40]
CNF@Ni-Co LDH	1–2000 μM	0.03 μM	[41]
NiCo_2_O_4_/EGF	0.0005–3.571 mM	0.167 μM	[42]
Ni/CNF	2 μM–5 mM	0.57 μM	[43]
CA/CS	5.0 μM–0.75 mM	4.8 μM	[44]
CS/GO	0.05–20 mM	0.02 mM	[45]
PEI/PVA	2–8 mM, 10–30 mM	0.3 mM	[46]
SnO_2_ multiporous NFs	0.5–5 mM	0.05 mM	[47]
Cellulose/β-CD	0–1 mM	9.35 × 10^−5^ M	[48]

LOD—limit of detection, PAN—polyacrylonitrile, PPy—polypyrrole, PPy3COOH—pyrrole-3-carboxylic acid, CNF—carbon nanofibers, AuNPs—gold nanoparticles, GO—graphene oxide, LDH—layered double hydroxide, EGF—electrospun graphitic nanofiber film, CA—cellulose acetate, CS—chitosan, PEI—poly(ethyleneimine), PVA—poly(vinyl alcohol), β-CD—β-cyclodextrin.

**Table 2:** Nanofiber-based sensors for electrochemical biomolecules detection.

Analyte	Sensor	Linear Range	LOD	Ref.
Dopamine	PANi/CQDs	10–90 μM	0.1013 μM	[49]
Dopamine	OLC-CNF	-	1.42 μM	[50]
Streptavidin	PS-b-PMMA	10 fg/mL–10 ng/mL	0.37 fg/mL	[51]
25-OHD_3_	CA	10–100 ng/mL	10 ng/mL	[52]
LD and UA	MoS_2_ NSA/CNF	1–60 μM	1 μM	[54]
MUC1 cancer marker	Honey/PVA + AuNPs + MWCNTs	5–115 nM	2.7 nM	[55]
Her-2 cancer marker	Ab/MWCNT/Cys/	5–80 ng/mL	0.45 ng/mL	[56]
	AuNP/CNF			
Nicotine	MWCNTs/CS	0.1–100 μM	30 nM	[57]
Progesterone	GQDs-NiO-Au NFs/MWCNTs	0.01–1000 nM	1.86 pM	[58]
ZEN	PAN	5–30 nM 60–100 nM	1.66 nM	[59]
OTA	Silanized cellulose	0.002–2 ng/mL	0.81 pg/mL	[60]
Malathion	rGO/PA6/PPy	0.5–20 μg/mL	0.8 ng/mL	[61]
VCT	CNF	10^−13^–10^−5^ g/mL	0.12 pg/mL	[62]
CEA	Honey/PVA + AuNPs + MWCNTs	0.4–125 ng/mL	0.09 ng/mL	[63]
COX-2	polyaniline	-	0.01 pg/mL	[64]
LD	Graphene/CNF	1–60 μM	1 μM	[65]

NFs—nanofibers, LOD—limit of detection, PANi—polyaniline, CQDs—carbon quantum dots, OLC—onion-like carbon, CNF—carbon nanofibers, PS-b-PMMA—poly(styrene-block-methyl-methacrylate), 25-OHD_3_—25-hydroxy vitamin D_3_, CA—cellulose acetate, LD—levodopa, UA—uric acid, NSA—nanosheet array, PVA—poly(vinyl alcohol), AuNPs—gold nanoparticles, MWCNTs—multiwalled carbon nanotubes, Ab—-antibody, Cys—cysteamine, CS—chitosan, GQDs—graphene quantum dots, ZEN—zearalenone mycotoxin, PAN—polyacrylonitrile, OTA—ochratoxin A, rGO—reduced graphene oxide, PA6—polyamide 6, PPy—polypyrrole, VCT—Vibrio cholerae toxin, CEA—carcinomaembryonic antigen, COX-2—cyclooxygenase-2.

**Table 3:** Nanofiber-based sensors for electrochemical detection of various drugs.

Analyte	Sensor	Linear Range	LOD	Ref.
Penicillin	AuNPs/CNF	1–400 ng/ml	0.6 ng/ml	[66]
Colchicine	MIL/CuO/CNF	1–100 nM	0.25 nM	[67]
APAP	SnO_2_-CNF	0.5–700 μM	0.086 μM	[68]
p-HAP		0.2–50 μM	0.033 μM	
Morphine	Magnetic NFs	0.033–245 μM	1.9 nM	[69]
Methotrexate	CuCr_2_O_4_/CuO	0.1–300 μM	25 nM	[70]
Tramadol	CNF	0.05–100 nM	0.016 nM	[71]
Metronidazole	CNF-NiCo-LDH	3–57 nM	0.13 nM	[72]

NFs—nanofibers, LOD—limit of detection, AuNPs—gold nanoparticles, CNF—carbon nanofibers, MIL—magnetic ionic liquid, APAP—acetaminophen, p-HAP—p-hydroxyacetophenone, LDH—layered double hydroxide.

**Table 4:** Nanofiber-based sensors for electrochemical metal ions detection.

Analyte	Sensor	Linear Range	LOD	Ref.
Hg^2+^	PES/QDs	0.1–150 nM	0.02 nM	[77]
Hg^2+^	AuNPs/PtNPs/CNF	1 fM–1 μM	0.33 fM	[78]
Hg^2+^	CNW/GO/PA6	2.5–200 μM	0.52 μM	[79,80]
Cd^2+^	ZIF-8NPs/N-doped CNF	2–100 μg/L	1.11 μg/L	[81]
Pb^2+^		1–100 μg/L	0.72 μg/L	
Pb^2+^	L-Cys/ZnO	10–140 μg/L	0.397 μg/L	[82]
As^3+^	AuNPs/PANi/Fe-CNF	0.07–5.34 μM	6.67 nM	[83]

NFs—nanofibers, LOD—limit of detection, PES—polyethersulfone, QDs—quantum dots, AuNPs—gold nanoparticles, PtNPs—platinum nanoparticles, CNF—carbon nanofibers, CNW—carbon nanowhiskers, GO—graphene oxide, PA6—polyamide 6, L-Cys—L-cysteine.

**Table 5:** Nanofiber-based sensors for optical metal ions detection.

Analyte	Sensor	LOD	Ref.
Hg^2+^	RIM/polyurethane	-	[84]
Hg^2+^	PVA/TEOS/S	0.018 ng/ml	[85]
Fe^3+^	FPN/PCL	2.94 nM	[86]
Fe^3+^	PASP	0.1 mg/mL	[87]
Cu^2+^		0.3 mg/ml	
Cu^2+^	RBD/CA	0.28 mM	[88]
Cu^2+^	QDs/PEI/PVDF	2 μM	[89]
Cu^2+^	QDs/PA6	10 μM	[90]

NFs—nanofibers, LOD—limit of detection, RIM—rhodamine derivative with N-methyl imidazole unit, PVA—poly(vinyl alcohol), TEOS/S—tetraethyl orthosilicate/carbazole-based Schiff base, FNP—4,4′-fluoresceinoxy bisphthalonitrile, PCL—polycaprolactone, PASP—poly(aspartic acid), RBD—rhodamine B derivative, CA—cellulose acetate, QDs—quantum dots, PEI—polyethylenimine, PVDF—poly(vinylidene fluoride), PA6—polyamide 6.

**Table 6:** Nanofiber-based sensors for optical detection of various molecules.

Analyte	Sensor	LOD	Ref.
Thrombin	DNA/PS	1 pM	[91]
Dopamine	CQDs/PANi	0.0801 µM	[49]
Dopamine	AuNPs/PET	0.5 µM	[92]
Pesticides	AChE/PVA, IA/PVA	0.02 mg/L	[93]
Kanamycin	cDNA/AuNPs/A/GA/CA	2.5 nM	[94]
Biothiols	DA-Hg-DA/PEO	-	[95]
Riboflavin, pH	Nanorods/Ag@SiO_2_/PS	0.03 µM	[96]
Bacteria	HCy + PU, PU/PVP, PU/PEG	10^5^ CFU/cm^2^,	[97]
		10^6^ CFU/cm^2^	

LOD—limit of detection, PS—polystyrene, CQDs—carbon quantum dots, PANi—polyaniline, AuNPs—gold nanoparticles, PET—poly(ethylene terephthalate), AChE—acetylcholinesterase, PVA—poly(vinyl alcohol), IA—indolyl acetate, A—aptamer, GA—glutamic acid, CA—cellulose acetate, DA-Hg-DA—mercury-complexed, pyridine-containing polydiacetylene, PEO—polyethyleneoxide, HCy—hemicyanine based chromogenic probe, PU—polyurethane, PVP—polyvinylpyrrolidone, PEG—polyethylene glycol

## Data Availability

Not applicable.
